# Effects of Erythromycin on Osteoclasts and Bone Resorption via DEL-1 Induction in Mice

**DOI:** 10.3390/antibiotics10030312

**Published:** 2021-03-17

**Authors:** Hikaru Tamura, Tomoki Maekawa, Hisanori Domon, Takumi Hiyoshi, Satoru Hirayama, Toshihito Isono, Karin Sasagawa, Daisuke Yonezawa, Naoki Takahashi, Masataka Oda, Takeyasu Maeda, Koichi Tabeta, Yutaka Terao

**Affiliations:** 1 Division of Microbiology and Infectious Diseases, Graduate School of Medical and Dental Sciences, Niigata University, Niigata 951-8514, Japan; h-tamura@dent.niigata-u.ac.jp (H.T.); hiyoshi@dent.niigata-u.ac.jp (T.H.); shirayama@dent.niigata-u.ac.jp (S.H.); tisono@dent.niigata-u.ac.jp (T.I.); k-sasagawa@dent.niigata-u.ac.jp (K.S.); terao@dent.niigata-u.ac.jp (Y.T.); 2 Center for Advanced Oral Science, Graduate School of Medical and Dental Sciences, Niigata University, Niigata 951-8514, Japan; maedat@dent.niigata-u.ac.jp; 3 Division of Periodontology, Graduate School of Medical and Dental Sciences, Niigata University, Niigata 951-8514, Japan; takahashi-n@dent.niigata-u.ac.jp (N.T.); koichi@dent.niigata-u.ac.jp (K.T.); 4 Division of Oral Science for Health Promotion, Graduate School of Medical and Dental Sciences, Niigata University, Niigata 951-8514, Japan; yonezawa01@dent.niigata-u.ac.jp; 5 Department of Microbiology and Infection Control Sciences, Kyoto Pharmaceutical University, Yamashita 607-8414, Japan; moda@mb.kyoto-phu.ac.jp

**Keywords:** macrolides, periodontitis, osteoclastogenesis, DEL-1

## Abstract

Macrolides are used to treat various infectious diseases, including periodontitis. Furthermore, macrolides are known to have immunomodulatory effects; however, the underlying mechanism of their action remains unclear. DEL-1 has emerged as an important factor in homeostatic immunity and osteoclastogenesis. Specifically, DEL-1 is downregulated in periodontitis tissues. Therefore, in the present study, we investigated whether the osteoclastogenesis inhibitory effects of erythromycin (ERM) are mediated through upregulation of DEL-1 expression. We used a ligature-induced periodontitis model in C57BL/6Ncrl wild-type or DEL-1-deficient mice and in vitro cell-based mechanistic studies to investigate how ERM inhibits alveolar bone resorption. As a result of measuring alveolar bone resorption and gene expression in the tooth ligation model, ERM treatment reduced bone loss by increasing DEL-1 expression and decreasing the expression of osteoclast-related factors in wild-type mice. In DEL-1-deficient mice, ERM failed to suppress bone loss and gene expression of osteoclast-related factors. In addition, ERM treatment downregulated osteoclast differentiation and calcium resorption in in vitro experiments with mouse bone marrow-derived macrophages. In conclusion, ERM promotes the induction of DEL-1 in periodontal tissue, which may regulate osteoclastogenesis and decrease inflammatory bone resorption. These findings suggest that ERM may exert immunomodulatory effects in a DEL-1-dependent manner.

## 1. Introduction

Periodontitis is a disease characterized by inflammation and bone resorption caused by dysbiotic oral microflora, which in turn affects the periodontal tissue, resulting in tooth loss [1]. Periodontopathic bacteria activate the inflammatory response via neutrophils excessively migrating into the periodontal tissue and promoting the production of inflammatory cytokines [2]. In addition, bone resorption is mediated by host immune cells and inflammatory cytokines, which promote osteoclast activity, particularly in states of inflammatory osteolysis such as periodontitis [3]. Therefore, in periodontitis treatment, in addition to the removal of dental biofilms, it is important to control inflammation and bone resorption by osteoclasts [4,5]. However, a method to control inflammation and bone resorption has not been established, and hence, it is necessary to search for a drug that can control inflammation and bone resorption. Therefore, various anti-inflammatory substances that suppress alveolar bone resorption have been reported as candidate substances for the treatment of periodontitis, for instance plant-derived ingredients with anti-inflammatory properties, such as polyphenols, hinokitiol, baicalin, and rice peptides [6,7,8,9,10].

Macrolides are a large family of protein synthesis inhibitors with broad-spectrum antibacterial activity and are used to treat various infectious diseases [11], such as skin infections, pneumonia, pyelonephritis, infectious enterocolitis, and periodontitis [12]. Furthermore, macrolides affect a wide range of immunological mechanisms, thereby providing immunomodulatory effects [13]. In respiratory infections such as pneumonia and rhinitis, macrolides are commonly used in anticipation of anti-inflammatory effects and used to inhibit bacterial pathogens in some cases [14,15]. Furthermore, macrolides are used to treat noninfectious diseases, such as chronic obstructive pulmonary disease (COPD) [16,17], diffuse panbronchiolitis [18], and certain forms of asthma [19].

In some clinical studies, beneficial clinical effects have been reported in the treatment of chronic periodontitis with macrolides [12]. Furthermore, azithromycin has been demonstrated to suppress osteoclast resorptive activity and osteoclast formation by regulating nuclear factor of activated T cells (NFATc1), a key osteoclast transcription factor [20]. However, the exact mechanism that suppresses the osteoclast differentiation effect of macrolides remains unclear.

We demonstrated that developmental endothelial locus-1 (DEL-1) is a protein that is induced by macrolides and exerts immunomodulatory effects such as suppression of neutrophil migration [21]. DEL-1 has emerged as an important factor in neutrophil regulation in the context of both the initiation and resolution of inflammation [22,23,24]. In addition, DEL-1 affects osteoclasts by regulating their differentiation. Mechanistically, DEL-1 was reported to inhibit the expression of NFATc1 in a Mac-1 integrin-dependent manner [25]. We recently reported that erythromycin (ERM) exerts its immunomodulatory effects by regulating the local homeostatic factor DEL-1 and reduces bone resorption by inhibiting excessive infiltration of neutrophils in a mouse model [21]. However, it is not clear whether ERM-induced DEL-1 actually acts on osteoclasts. In this study, we hypothesized that ERM-induced DEL-1 directly regulates osteoclasts and is involved in the suppression of alveolar bone resorption. The effects of ERM and DEL-1 on osteoclast differentiation and bone resorption activity were investigated both in vitro and in vivo using wild-type (WT) or DEL-1-deficient mice.

## 2. Results

### 2.1. Oral Administration of Antibacterial Drugs Significantly Suppresses Periodontal Bone Loss Induced by Tooth Ligation 

**Figure 1:**
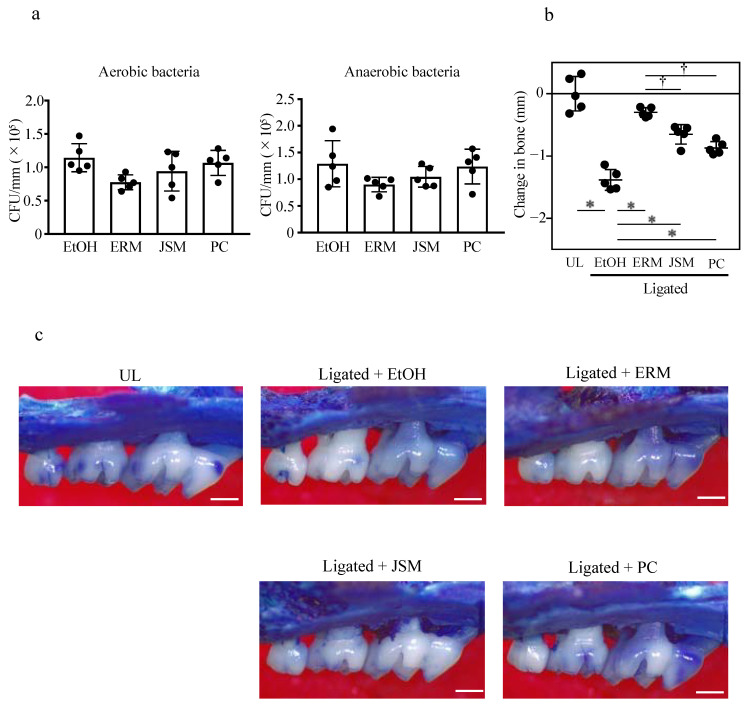
Peritoneal injection of antibiotics inhibits bone resorption in ligature-induced periodontitis. Periodontal bone loss was induced by ligating the maxillary second molar. The unligated (UL) group was set as the baseline control. Groups of mice were administered intraperitoneally with 20% ethanol (EtOH; control), penicillin (PC), josamycin (JSM), or erythromycin (ERM). (**a**) Number of aerobic and anaerobic bacteria attached to the silk ligature. (**b**) The distance from the cement-enamel junction to the pinnacle of the alveolar bone was measured. Negative values (in mm) indicate bone loss relative to the UL control. (**c**) Representative images of mouse maxillary bones from the indicated groups (scale bars, 0.5 mm). * *p* < 0.05, compared to the EtOH group; ^†^ *p* < 0.05, compared to the ERM group, means ± SD (*n* = 5 per group).

We previously established a mouse model of ligature-induced periodontitis that could mimic dysbiosis of the human biofilm in periodontitis [26,27,28]. To examine whether ERM, josamycin (JSM), and penicillin (PC) inhibit inflammatory alveolar bone resorption in this model, we administered antibacterial drugs orally to the mice. This mouse model of periodontitis showed significantly increased alveolar bone loss compared to the unligated (UL) group (Figure 1a). Figure S1a shows that in comparison to the UL group, alveolar bone resorption was significantly suppressed by treatment with any of the tested antibacterial drugs. In addition, treatment with antibacterial drugs significantly decreased the number of aerobic and anaerobic bacteria attached to the ligature compared to that of the distilled water group (Figure S1b,c). These findings clearly indicate that the antibacterial property suppressed alveolar bone resorption in periodontitis. 

### 2.2. Intraperitoneal Injection of Erythromycin Significantly Suppresses Periodontal Bone Loss Induced by Tooth Ligation

**Figure 2:**
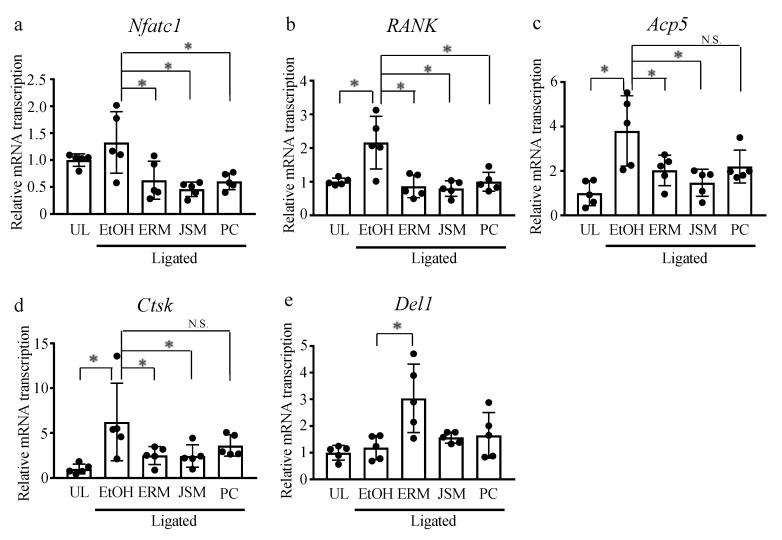
Effect of antibacterial drugs on the transcription of osteoclast-related factors in the gingiva from ligature-induced periodontitis model mice. (**a**–**e**) Real-time qPCR was performed to quantify the mRNA transcription levels of osteoclast-related factors and *Del1*. * *p* < 0.05 compared to the phosphate-buffered saline (PBS) group, means ± SD (*n* = 5 per group). N.S., not significant.

Based on these results, the experimental method was changed to intraperitoneal administration, which may be suitable for evaluating the anti-inflammatory effect of these antibiotics. In contrast to oral administration, intraperitoneal injection of these antibiotics did not significantly affect the number of aerobic and anaerobic bacteria attached to the ligature (Figure 1a). However, these antibiotics significantly reduced bone loss compared to the ethanol (EtOH) group. Additionally, the ERM treatment significantly reduced bone loss compared to the PC and JSM treatment groups (Figure 1b,c). Compared to the EtOH group, ERM, JSM, and PC treatment significantly decreased the gene expression of *Nfatc1* and *RANK*, which are osteoclast differentiation-related factors in the gingiva (Figure 2a,b). Furthermore, ERM and JSM treatment significantly downregulated the expression of bone resorption activity-related factors (*Acp5*, *Ctsk*) (Figure 2c,d). Interestingly, ERM treatment significantly upregulated *Del1* expression, compared to the EtOH group (Figure 2e).

### 2.3. ERM Treatment Reduces the Number of Osteoclasts in Periodontal Ligament Tissue

**Figure 3:**
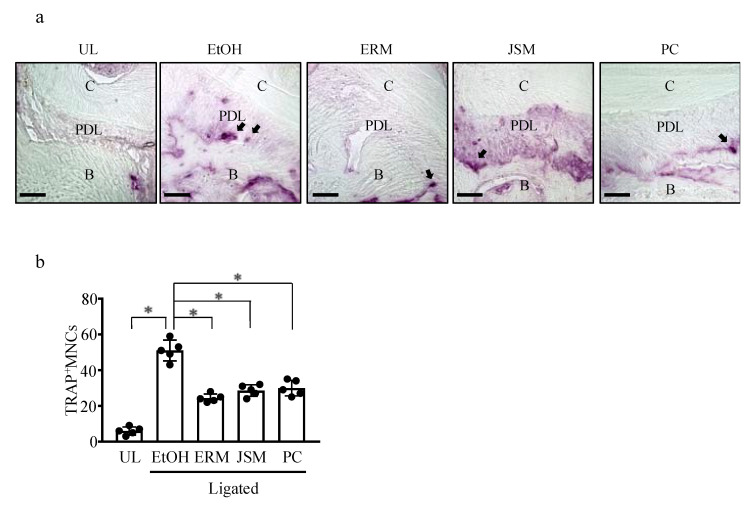
Erythromycin inhibits ligature-induced osteoclast differentiation. (**a**,**b**) Frozen maxillae sections were stained with tartrate-resistant acid phosphatase (TRAP). (**a**) Representative images obtained by optical microscopy are shown; arrows indicate TRAP^+^ osteoclasts. C: cementum; PDL: periodontal ligament; B: alveolar bone. (**b**) TRAP-reactive multinucleated cells (MNCs) were counted in five coronal sections of ligated sites of each mouse. Scale bars, 50 µm. * *p* < 0.05 compared to ligated + ethanol (EtOH) group, means ± SD (*n* = 5 per group).

Figure 3a,b show that the number of tartrate-resistant acid phosphatase-reactive (TRAP^+^) mononuclear cells (MNCs) around the second molar was significantly increased in the ligated group compared to the unligated control group. Consistent with the bone loss measurements, intraperitoneal injection of ERM, JSM, and PC caused a significant reduction in the number of TRAP^+^ MNCs in bone tissue sections (Figure 3a,b). 

### 2.4. ERM Treatment Does Not Affect Alveolar Bone Resorption in Del1^−/−^ Mice

**Figure 4:**
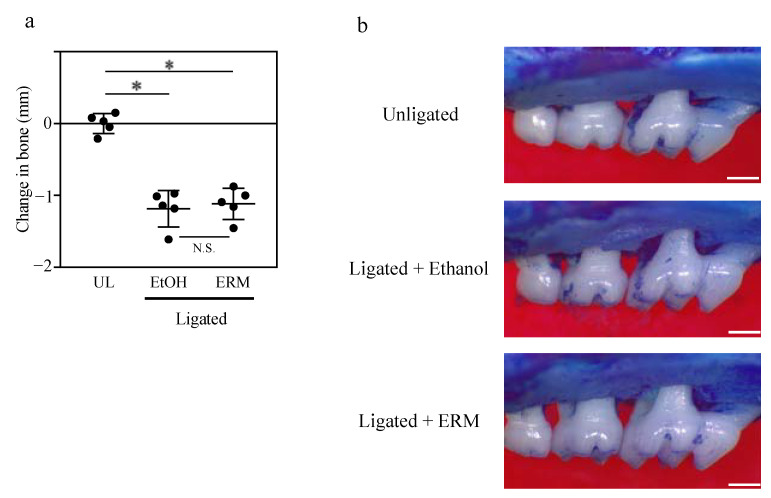
Erythromycin fails to inhibit bone resorption in *Del1^−/−^* mice. Periodontal bone loss was induced in *Del1^−/−^* mice. The unligated (UL) group was set as the baseline control. Groups of mice after tooth ligation were administered intraperitoneally with 20% ethanol (EtOH; control), penicillin (PC), josamycin (JSM), or erythromycin (ERM). (**a**) The distance from the cement enamel junction to the pinnacle of the alveolar bone was measured. Negative values (in mm) indicated bone loss relative to the UL control. (**b**) Representative images of mouse maxillary bones from the indicated groups (scale bars, 0.5 mm). * *p* < 0.05, compared to the indicated group, means ± SD (*n* = 5 per group). N.S., not significant.

**Figure 5:**
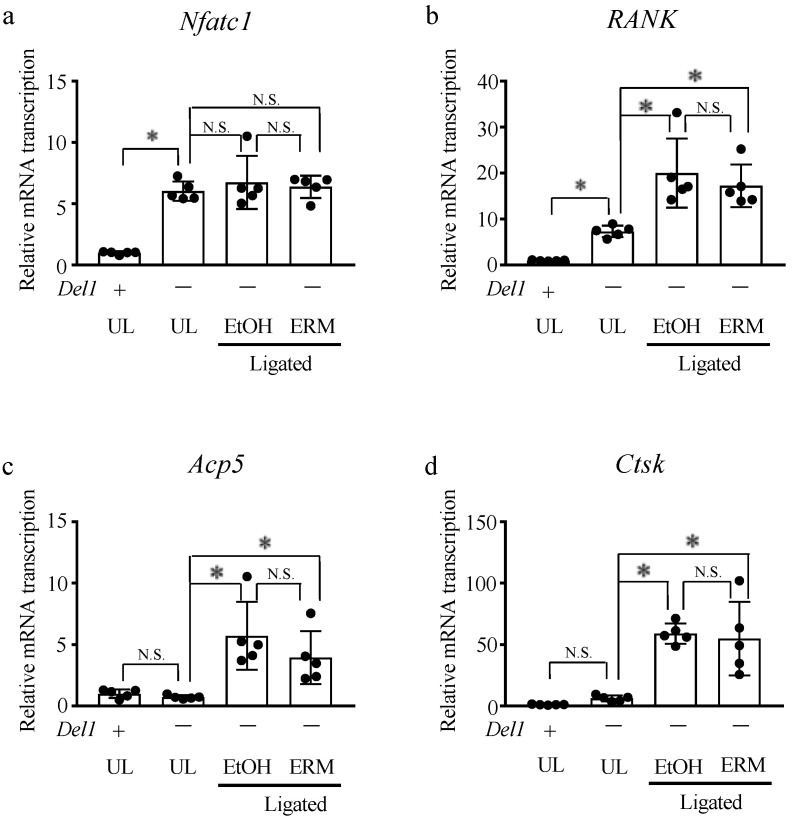
Effect of erythromycin on the transcription of osteoclast-related factors in *Del1*^−/−^ mice. (**a**–**d**) Real-time qPCR was performed to quantify the mRNA transcription levels of osteoclast-related factors in *Del1*^−/−^ mice. * *p* < 0.05 compared to the indicated groups, means ± SD (*n* = 5 per group). N.S., not significant.

Based on these findings, we hypothesized that ERM treatment inhibits alveolar bone resorption in a DEL-1-dependent manner. Therefore, we further examined whether ERM suppresses ligature-induced alveolar bone resorption in *Del1^−/−^* mice. In contrast to WT mice, ERM failed to reduce bone loss in *Del1^−/−^* mice (Figure 4a,b). Interestingly, there was no difference in *Nfatc1* and *RANK* gene expression between the EtOH group and ERM-treated *Del1^−/−^* mice (Figure 5a,b). Additionally, the expression of these genes was significantly upregulated in the UL group of *Del1^−/−^* mice compared to that in the UL group of WT mice (Figure 5a,b). The expression of *Acp5* and *Ctsk* was not upregulated in unligated *Del1^−/−^* mice, and the expression of these genes did not affect the ERM treatment (Figure 5c,d). These results suggest that DEL-1 is significantly involved in osteoclast differentiation and that ERM may exert its inhibitory effect in a DEL-1-dependent manner.

**Figure 6:**
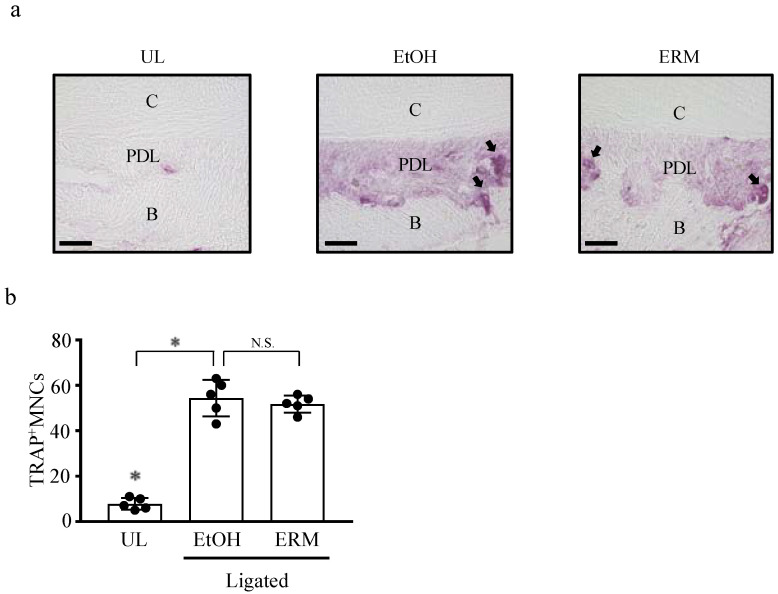
Erythromycin fails to inhibit ligature-induced osteoclast differentiation in *Del1^−/−^* mice. (**a**,**b**) Frozen maxillae sections were stained with tartrate-resistant acid phosphatase (TRAP). (**a**) Representative images obtained by optical microscopy are shown; arrows indicate TRAP^+^ osteoclasts. (**b**) TRAP-reactive multinucleated cells (MNCs) were counted in five coronal sections of ligated sites of each mouse. Scale bars, 50 µm. * *p* < 0.05 compared to ligated + ethanol (EtOH) group, means ± SD (*n* = 5 per group). N.S., not significant.

Although the number of TRAP^+^ MNCs around the second molar was significantly higher in the ligated + EtOH group than in the UL group in *Del1^−/−^* mice, ERM treatment did not decrease the number of TRAP^+^ MNCs (Figure 6a,b). These results suggest that ERM may suppress osteoclast differentiation by inducing DEL-1.

### 2.5. ERM Treatment Suppresses Osteoclast Differentiation in Bone Marrow-Derived Macrophages

**Figure 7:**
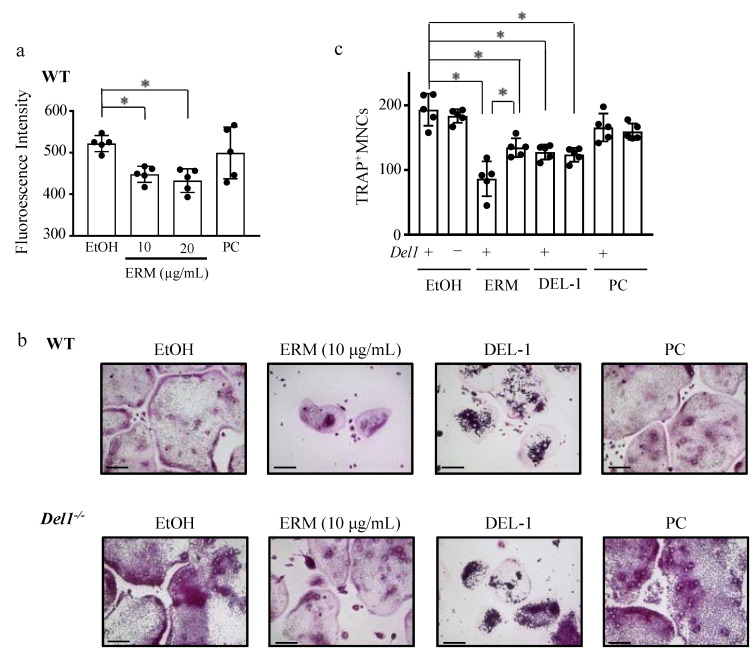
Erythromycin suppresses osteoclast differentiation in vitro in a DEL-1-dependent manner in wild-type mice. RANKL-induced osteoclastogenesis and bone resorption activity were determined in wild-type (WT) or *Del1*^−/−^ mouse bone marrow-derived osteoclast precursors in the presence of ethanol (EtOH; control), erythromycin (ERM; 10, 20 μg/mL), penicillin (PC; 5 units/mL), and DEL-1-Fc (DEL-1; 5 μg/mL). (**a**) Osteoclasts were cultured on a fluoresceinamine-labeled sodium chondroitin poly-sulfate/calcium phosphate (FACPS/CaP)-coated plate, and the absorption activity was examined by measuring the fluorescence intensity of the culture supernatant. (**b**) Representative images of TRAP^+^ osteoclasts obtained by optical microscopy are shown. Scale bars, 100 μm. (**c**) Cells were stained for tartrate-resistant acid phosphatase (TRAP) to detect osteoclasts, and TRAP-reactive MNCs were counted. * *p* < 0.05, compared to the indicated group, means ± SD (*n* = 5 per group).

We next examined the effect of ERM on osteoclast differentiation of mouse bone marrow-derived macrophages (BMM) in vitro. Figure S2a,b show that treatment of WT-BMM with ERM significantly decreased TRAP^+^ MNCs in a dose-dependent manner. Subsequently, we investigated the effect of ERM on the bone-resorbing activity of WT-BMM using fluoresceinamine-labeled sodium chondroitin poly-sulfate/calcium phosphate (FACPS/CaP)-coated plates. ERM treatment decreased the fluorescence intensity of the culture supernatant (Figure 7a). Neither ERM nor PC showed cytotoxicity toward BMMs at the tested concentrations (Figure S3). We next investigated the effect of ERM and recombinant DEL-1 on the osteoclastogenesis of *Del1^−/−^* mice. In both WT and *Del1^−/−^* mice, ERM and DEL-1 treatment significantly decreased the number of TRAP^+^ MNCs. However, the effect was significantly stronger in the BMM of the WT mice than in the BMM of the *Del1^−/−^* mice. (Figure 7b,c). Consequently, ERM may partly suppress osteoclast differentiation in a DEL-1-dependent manner.

## 3. Discussion

Macrolides have the characteristic structural features of a macrocyclic lactone ring to which various deoxy sugars, generally cladinose and desosamine, are attached [29]. Furthermore, macrolide antibiotics are classified into several types according to their structural differences; the most common substances are 14-, 15-, and 16-member rings. Typical drugs include ERM, clarithromycin (CAM), and roxithromycin (RXM) for the 14-membered rings, azithromycin (AZM) for the 15-membered rings, and spiramycin and JSM for the 16-membered rings. ERM, RXM, CAM, and AZM have been reported to have immunomodulatory effects, whereas the 16-membered ring JSM has no reports of such effects [13,30]. Therefore, JSM was used in our study as a negative control together with PC. Indeed, ERM exerted a stronger bone resorption inhibitory effect than JSM. In addition, several effects of 14-membered ring macrolides on inflammatory bone disease have been reported; 14-membered ring macrolides have been shown to be effective in the treatment of rheumatoid arthritis [31,32]. Furthermore, there are several reports that ERM is effective in preventing postoperative infection after artificial joint replacement by suppressing osteoclast differentiation [33,34]. However, little is known about the immunomodulatory and osteoclast-inhibitory mechanisms of macrolides. In this study, we showed that DEL-1 plays an important role in the immunomodulatory effect of ERM in a mouse model of periodontitis. Although several papers suggested that the composition of subgingival bacteria is a key factor of periodontitis in both human and mouse models [35,36,37], in the present study, we evaluated the total number of bacteria. We revealed that intraperitoneal injection of ERM notably decreased alveolar bone loss without a significant reduction in the number of viable bacteria attached to the ligature in a WT mouse model of periodontitis. These results indicate that ERM may suppress alveolar bone resorption via an immunomodulatory effect by inducing DEL-1, which regulates osteoclast differentiation in the ligature-induced periodontitis mouse model, although not excluding other models. 

Various antibacterial drugs are used to treat periodontitis, including beta-lactam derivatives, macrolides, nitroimidazole, and tetracyclines. Based on the results of meta-analyses, beneficial therapeutic effects of macrolides with immunomodulatory effects have been reported [38,39,40]. It has been reported that a subantibiotic dose of the 15-membered macrolide AZM attenuates alveolar bone loss in a rat model of experimental periodontitis [41]. The suppression of alveolar bone resorption by ERM treatment in the mouse periodontitis model in the present study is consistent with the mentioned findings. Macrolide antibiotics have different characteristics from other antibiotics in the concentration of inflamed tissue. The administered macrolides containing ERM are present in high concentrations in the blood, extracellular interstitial fluid, and intracellularly [42]. Moreover, inflammatory cells accumulate macrolides and transport them to tissues that release chemoattractant molecules. Inflammatory cells that accumulate macrolides release macrolides by activating bacterial components in the inflamed tissue [43,44]. These facts indicate that high levels of ERM in the inflamed tissue may affect surrounding cells such as endothelial cells, osteolineage cells, and certain macrophage subsets in the periodontal tissue. In a previous study, we reported that ERM modulates DEL-1 expression by activating GHSR/JAK2 signaling in vascular endothelial cells [21]. Consequently, it was suggested that ERM may exert an inhibitory effect on osteoclast differentiation by increasing the expression of DEL-1 in the cells of the periodontal tissue.

Several side effects and issues with resistant bacteria have been reported with the use of erythromycin in periodontal treatment. Among the reported side effects with erythromycin are gastrointestinal problems, skin allergies, and central nervous system problems. In addition, administration of erythromycin should be avoided in patients with renal impairment and in patients taking oral digoxin [45,46]. Although the cause remains unclear, several hypotheses have been suggested, including changes in the colonic bacterial flora and in the host immunity [47]. Increased levels of resistance to erythromycin between clinical and symbiotic isolates have been a challenge for a long time. Furthermore, these increases were reported to correlate with increased use of this class of antibiotics. Specifically, it has been reported that 7% of the culturable microflora from 20 samples is erythromycin resistant and carries the erythromycin resistance gene [48]. In addition, it was reported that the erythromycin resistance genes erm (B) and erm (F) were detected by PCR in 58 of 100 clinical oral Prevotella isolates [49]. Long-term use of macrolide antibiotics, which are expected to have immunomodulatory effects, may promote the growth of drug-resistant bacteria. Therefore, the development of macrolide antibiotics that have no antibacterial activity is expected [50]. We expect that the present study’s partial elucidation of the immunomodulatory mechanism of macrolide antibacterial agents will lead to their development in the future.

DEL-1 is a 52 kDa protein that is secreted from various tissue-resident cells such as endothelial cells, osteolineage cells, and certain macrophage subsets [23,51,52,53]. DEL-1, which is characterized by functional versatility and homeostatic properties, affects cells and the extracellular matrix in most tissues [54,55,56,57]. For instance, it has been reported that the anti-neutrophil mobilization action of endothelial cell-derived DEL-1 is associated with various respiratory diseases, including allergic asthma, pulmonary fibrosis, and melanoma lung metastasis [58,59,60]. Regarding the effects on osteolineage cells, it has been reported that DEL-1 suppresses the differentiation of and bone resorption by osteoclasts [25]. Furthermore, there are reports that DEL-1 effects on osteoblasts promote bone formation [61]. Thus, it is possible that in our study, ERM strongly suppressed bone resorption by increasing the expression of DEL-1 in the periodontal tissue. Indeed, we previously reported that RvD1 suppresses alveolar bone resorption by inducing DEL-1 [62]. Furthermore, the increased expression of *Nfatc1* in the healthy gingival tissue of *Del1^−/−^* mice also indicates the strength of the association between DEL-1 and bone resorption. An important substance involved in osteoclast differentiation is *Nfatc1*, a master regulator of osteoclast formation induced by a nuclear factor-κB ligand receptor activator (RANKL). In addition, transcription factors that negatively regulate *Nfatc1* activity are known, such as the interferon regulatory factor-8 (IRF-8), B-cell lymphoma 6 (Bcl6), and v-Maf myoaponeurotic fibrosarcoma oncogene family member protein B (MafB) [63]. DEL-1 suppresses transcription of NFATc1 by binding to Mac-1 and increasing Bcl6 expression. Moreover, DEL-1 with its RGD motif suppresses bone resorption by binding to αvβ3, which is important for the bone resorption function of osteoclasts [25,64]. Consequently, DEL-1 negatively regulates osteoclast differentiation.

## 4. Materials and Methods

### 4.1. Reagents

DEL-1 was purchased from R&D Systems (Minneapolis, MN, USA). The following antibacterial drugs were used for the treatment of mice: ERM (FUJIFILM Wako Pure Chemical Corporation, Tokyo, Japan), JSM (Sigma-Aldrich, St. Louis, MO, USA), and PC (Meiji Seika Pharma Co., Ltd., Tokyo, Japan). Each substance was dissolved in 80% phosphate-buffered saline (PBS) and 20% ethanol.

### 4.2. Murine Tooth Ligating Model

All animal experiments were approved by the Institutional Animal Care and Use Committee of Niigata University (SA00181). We purchased C57BL/6Ncrl mice from Charles River Laboratories (Japan, Inc., Kanagawa, Japan). The mice were maintained in individually ventilated cages and provided with sterile food and water ad libitum under specific pathogen-free conditions. They were used for experiments at the age of 10–12 weeks. We commissioned Setsuro Tech (Tokushima, Japan) to create a C57BL/6Ncrl *Del1^−/−^* mouse strain using the GEEP method [65,66]. CRISPR RNA was designed (*Del1* up crRNA: CTGGCTTTGGGCGCCCCCGG; protospacer adjacent motif (PAM): CGG; *Del1* down crRNA: GGGGTGCCCCAGTTCGGCAA; PAM:AGG) as described by Choi [24].

To establish the model, a 5–0 silk ligature (Akiyama MEDICAL MFG. CO., LTD., Tokyo, Japan) was tied around the maxillary second molar to generate ligature-induced periodontitis [67]. Antibacterial drugs (ERM; 100 mg/kg body weight, PC; 10,000 unit/kg body weight, JM; 100 mg/kg body weight) or 20% EtOH were administered intraperitoneally once a day for 9 days in the intervention experiments. The mice were euthanized 9 days after ligating the second molar. The gingiva was dissected and processed for real-time quantitative PCR (qPCR) measurements. We analyzed osteoclast-related factors and *Del1* mRNA expression levels. A stereoscopic microscope (Leica Microsystems, Wetzlar, Germany) (35×) was used to assess morphologically the periodontal bone resorption of the defleshed maxilla. Specifically, the distance from the cement-enamel junction to the alveolar bone crest (CEJ-ABC) was measured at seven site-predetermined points on the ligated second molar and adjacent affected regions. The bone change was calculated by subtracting the sum of the CEJ-ABC values from the seven corresponding values in the unligated areas. Negative values (in mm) indicate bone loss relative to the baseline (unligated control).

### 4.3. Histologic Analysis

The following processes were performed to assess the standard histological and quantitative histomorphometric analyses. The maxillae of the tooth ligation model mice were collected 9 days after ligation and fixed in 4% paraformaldehyde PBS (Wako Pure Chemical Industries, Osaka, Japan) for 24 h. Subsequently, the samples were decalcified with Decalcifying Solution B (Wako Pure Chemical Industries) for 1 week at 4 °C. The specimens were then embedded in O.C.T. Compound (Sakura Finetek, Torrance, CA, USA), which was frozen in liquid nitrogen. Coronary sections were cut with a cryostat (Leica Biosystems, Wetzlar, Germany). The sections were stained with TRAP (Wako Pure Chemical Industries), and TRAP-reactive multinuclear giant cells (MNCs) were counted from five random coronal sections of the ligation sites from each mouse.

### 4.4. Cell Preparation and Culture

Bone marrow-derived cells were collected from the femurs and tibias of mice. After lysis of erythrocytes using RBC lysis buffer (Lonza, Basel, Switzerland), bone marrow-derived cells were cultured in 96-well plates (1.0 × 10^5^ per well) with recombinant murine macrophage colony-stimulating factor (M-CSF) (30 ng/mL; R&D Systems, Minneapolis, MN, USA) for 3 h in minimum essential medium eagle, alpha modification (α-MEM; FUJIFILM Wako Pure Chemical Corporation) with 10% fetal bovine serum (FBS) at 37 °C and 5% CO_2_. The nonadherent cell population was washed, and adherent cells were further cultured in α-MEM media supplemented with 10% FBS, 100 ng/mL recombinant soluble receptor activator of nuclear factor-kappa B ligand (RANKL; R&D Systems), and 100 ng/mL M-CSF in the presence or absence of ERM (1, 10, and 20 μg/mL) or PC (5 unit/mL). After 7 days, the cells were fixed and stained for TRAP using an acid phosphatase leukocyte diagnostic kit (Sigma-Aldrich), and TRAP-reactive multinucleated (≥3 nuclei) cells per well were counted. To determine the effect of DEL-1 on osteoclastogenesis, BMM from WT mice and *Del1^−/−^* mice were cultured in α-MEM containing M-CSF and RANKL in the presence or absence of 20% EtOH, ERM (10 μg/mL), DEL-1 (5 μg/mL), or PC (5 units/mL) for 1 week. Subsequently, the number of TRAP-reactive multinucleated (≥3 nuclei) cells per well was counted. FACPS or calcein solution (100 μg/mL) was added to CaP-coated 48-well plates (0.25 mL/well) and incubated at 37 °C for 2 h. The plates were washed twice with PBS and used for assays (FACPS/CaP-coated plates; PG Research Co., Ltd., Tokyo, Japan) [68]. Bone marrow cells were cultured in α-MEM media supplemented with 10% FBS in the presence of 100 ng/mL soluble recombinant RANKL (R&D Systems) and 100 ng/mL M-CSF to generate osteoclasts. The cells were treated with ERM (1, 10, and 20 μg/mL) or PC. After 7 days, the supernatant was collected, and the fluorescence intensity (Ex: 485 nm, Em: 535 nm) was measured using GloMax (Promega Corporation, Madison, WI, USA).

### 4.5. Quantitative Real-Time PCR

Total RNA was extracted from mouse maxillary palatal gingiva or BMMs using TRI reagent (Molecular Research Center, Inc., Cincinnati, OH) and quantified by spectrophotometry at 260 and 280 nm. The RNA was reverse-transcribed using SuperScript VILO Master Mix (Thermo Fisher Scientific, MA, USA). Quantitative PCR with the cDNA was performed according to the manufacturer’s protocol using the StepOnePlus real-time PCR system (Thermo Fisher Scientific). We analyzed data using the comparative CT (ΔΔCt) method. TaqMan probes, sense primers, and antisense primers for the expression of a housekeeping gene (*Gapdh*), along with *Nfatc1*, TNF receptor superfamily member 11a (Tnfrsf11a; *RANK*), acid phosphatase 5 (*Acp5*, encoding TRAP), cathepsin K (*Ctsk*), and EGF-like repeats and discoidin domains 3 (*Edil3*; *del1*, encoding DEL-1) were purchased from Thermo Fisher Scientific.

### 4.6. Statistical Analysis

We evaluated data by analysis of variance and one-way ANOVA with Tukey’s multiple comparisons test using GraphPad, Version 7.03 (GraphPad Software, Inc., La Jolla, CA, USA). A *p*-value of < 0.05 was considered statistically significant.

## 5. Conclusions

In conclusion, the in vivo and in vitro evidence suggests that ERM exerts an immunomodulatory effect by inducing DEL-1 and suppressing osteoclast differentiation. In particular, induced DEL-1 suppresses *Nfatc1* via Mac-1 and suppresses osteoclast differentiation [25]. Our study supports the use of ERM as an immunomodulatory drug for treating inflammatory bone resorption disease.

## Data Availability

All data are contained within the manuscript.

## Supplementary Materials

The following are available online at https://www.mdpi.com/2079-6382/10/3/312/s1, Materials and Methods S1: Supplementary Materials and Methods for supplementary figures. FigureS1: Oral administration of antibacterial drug failed to show immunomodulatory effects. Figure S2: Erythromycin dose-dependently suppresses osteoclast differentiation in vitro. Figure S3: Cytotoxicity of ERM or PC or JSM to osteoclasts.
