# Supplementary material for: Effects of Erythromycin on Osteoclasts and Bone Resorption via DEL-1 Induction in Mice
Source: Antibiotics (Basel). 2021 Mar 17;10(3):312. doi: 10.3390/antibiotics10030312 (PMC8002756; doi:10.3390/antibiotics10030312)
Supplement: Supplementary file 1 [file antibiotics-10-00312-s001.zip › Supplementary Materials/Materials and Methods S1.docx]

**Materials and Methods S1: S**upplementary Materials and Methods for supplementary figures.

1. Murine Tooth Ligation Model

To generate ligature-induced periodontitis, a 5–0 silk ligature (Akiyama MEDICAL MFG. CO., LTD., Tokyo, Japan) was tied around the maxillary second molar. Antibacterial drugs (ERM; 20 mg/kg body weight, PC; 10000 unit/kg body weight, JM; 20 mg/kg body weight), or distilled water (DW) were administered orally once a day for 7 days in the intervention experiments. The mice were euthanized 7 days after ligature placement.

2. Analysis of Bacteria Attached to the Ligature

The tube with the ligature was vortexed for 1 min 30 s. The PBS in this tube was diluted after agitation by 10-, 100-, 1000-, and 10000-fold. A volume of 100 μL of each dilution was inoculated on individual blood agar plates. Cells were cultured overnight in aerobic or anaerobic conditions at 37°C. The appropriate plate (1000-fold or 10000-fold dilution) suitable for counting colonies was selected for the analysis.

3. Cell Viability

BMM cells were seeded in FACPS/CaP-coated plates at a density of 1.0×10^5^ cells/well and incubated for 7 days. Cells were treated with ERM (1, 10, and 20 μg/mL), PC, or JSM for 7 days. Cell viability was assessed using the MTT assay, in which MTT (400 µg/mL) was directly added to the cultures, followed by incubation at 37°C and 5% CO_2_ in humidified air. Subsequently, the supernatant was aspirated, and 200 µL of lysis solution (90% isopropanol, 0.5% SDS, 0.04 N HCl, DW) were added to dissolve the formazan dye. The optical density (OD) was measured at 570 nm using a microplate reader. The mean OD of the control group was set as 1, and the experimental groups were compared to this control group.

**Supplementary figures S1-S3**

**Figure S1.** Oral administration of antibacterial drugs had no apparent immunomodulatory effects. Periodontal bone loss was induced by ligating the maxillary second molar. The unligated (UL) group was set as the baseline control. Groups of mice were administrated orally either distilled drinking water (DW; control) alone or with erythromycin (ERM), josamycin (JSM), or penicillin (PC). (a) The distance from the cement-enamel junction (CEJ) to the pinnacle of the alveolar bone was measured at seven predetermined points on the ligated second molar and the affected adjacent regions. Negative values (in mm) indicate bone loss relative to the baseline (unligated control). (**b**) Number of aerobic and anaerobic bacteria attached to the silk ligature. **p* < 0.05, compared to the ethanol (EtOH) group, means ± SD (*n* = 5 per group). N.S., not significant.

**Figure S2.** Erythromycin dose-dependently suppresses osteoclast differentiation in vitro. RANKL-induced osteoclastogenesis and bone resorption activity were determined in mouse bone marrow-derived osteoclast precursors in the presence of ethanol (EtOH; control), erythromycin (ERM; 1, 10, 20 μg/mL), or penicillin (PC; 5 units/mL). (**a)** Representative images of tartrate-resistant acid phosphatase (TRAP)^+^ osteoclasts obtained by optical microscopy are shown. Scale bars, 100 μm. (**b**) Cells were stained for TRAP to detect osteoclasts, and TRAP-reactive MNCs were counted. **p* < 0.05, compared to the indicated group, means ± SD (*n* = 5 per group).

**Figure S3.** Cytotoxicity of erythromycin (ERM), penicillin (PC), and josamycin (JSM) in osteoclasts. Cell viability was assessed using the MTT assay on fluoresceinamine-labeled sodium chondroitin poly-sulfate/calcium phosphate (FACPS/CaP)-coated plates. Cells were treated with ERM (1, 10, and 20 μg/mL), PC (5 unit/mL), or JSM (10 μg/mL) for 6 days. Means ± SD.
