# Supplementary figures and images for: Effects of Erythromycin on Osteoclasts and Bone Resorption via DEL-1 Induction in Mice
Source: Antibiotics (Basel). 2021 Mar 17;10(3):312. doi: 10.3390/antibiotics10030312 (PMC8002756; doi:10.3390/antibiotics10030312)

a

Ethanol

ERM 1  $\mu\text{g}$ ERM 10  $\mu\text{g}$ ERM 20  $\mu\text{g}$ 

PC

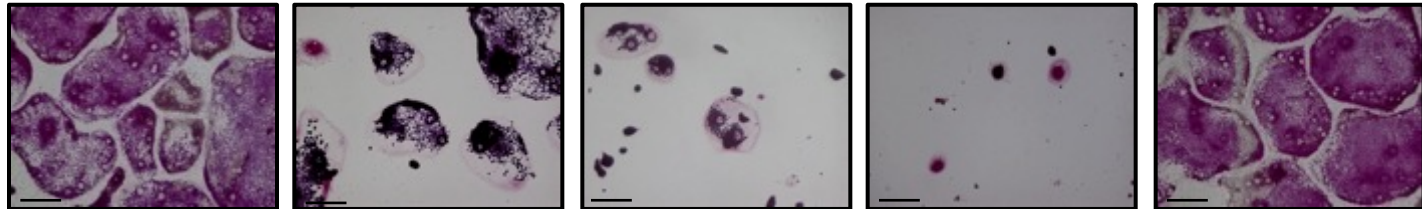

b

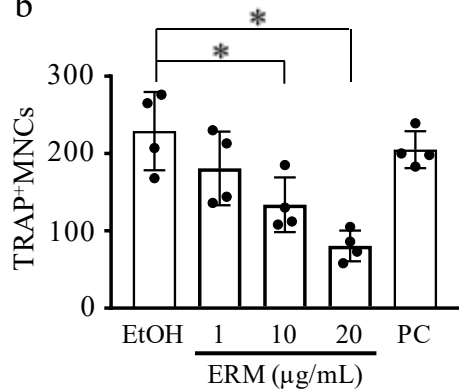

Supplement: Supplementary file 1 [file antibiotics-10-00312-s001.zip › Supplementary Materials/FigureS2.pdf]

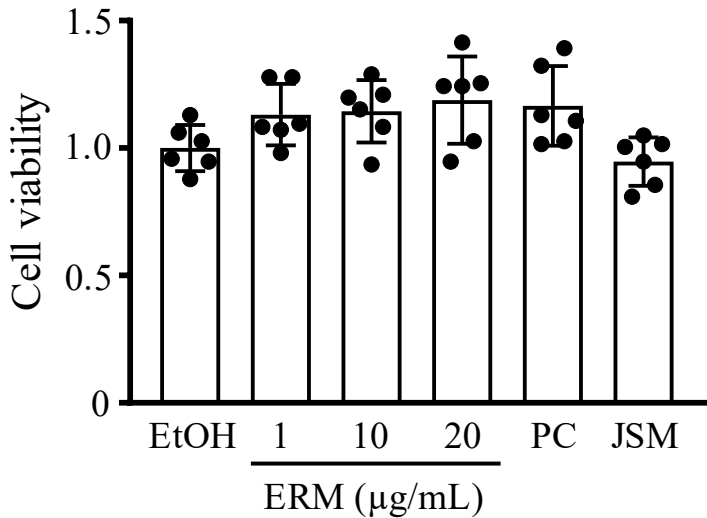

Supplement: Supplementary file 1 [file antibiotics-10-00312-s001.zip › Supplementary Materials/FigureS3.pdf]

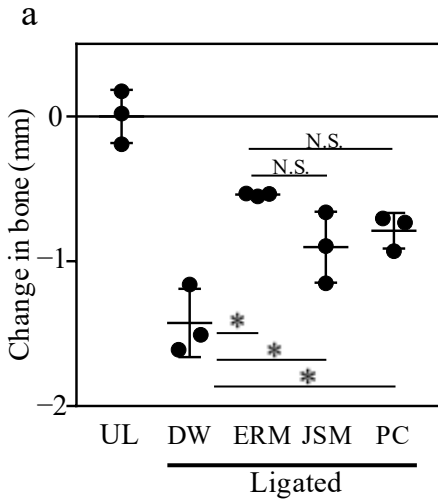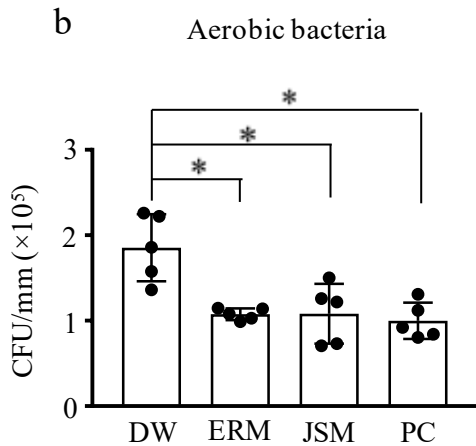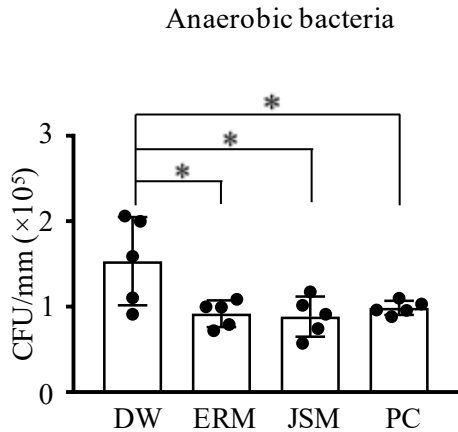

Supplement: Supplementary file 1 [file antibiotics-10-00312-s001.zip › Supplementary Materials/FigureS1.pdf]
